# LAMC2 mitigates ER stress by enhancing ER-mitochondria interaction via binding to MYH9 and MYH10

**DOI:** 10.1038/s41417-023-00680-5

**Published:** 2023-10-27

**Authors:** Dongdong Tong, Jun Zhou, Jing Zhou, Xiaofei Wang, Beibei Gao, Xiaoyi Rui, Liying Liu, QiaoYi Chen, Chen Huang

**Affiliations:** 1 Department of Cell Biology and Genetics, School of Basic Medical Sciences, Xi’an Jiaotong University Health Science Center, 710061 Xi’an, Shaanxi China; 2 Department of Pharmacology, School of Basic Medical Sciences, Health Science Center, Xi’an Jiaotong University, 710061 Xi’an, Shaanxi China; 3 Biomedical Experimental Center of Xi’an Jiaotong University, 710061 Xi’an, Shaanxi China; 4 Key Laboratory of Environment and Genes Related to Diseases, Xi’an Jiaotong University, Ministry of Education, 710061 Xi’an, China

**Keywords:** Cell biology, Oncogenes

## Abstract

Highly proliferative and metastatic tumors are constantly exposed to both intrinsic and extrinsic factors that induce adaptation to stressful conditions. Chronic adaptation to endoplasmic reticulum (ER) ER stress is common to many different types of cancers, and poses a major challenge for acquired drug resistance. Here we report that LAMC2, an extracellular matrix protein upregulated in many types of cancers, is localized in the ER of lung, breast, and liver cancer cells. Under tunicamycin-induced ER stress, protein level of LAMC2 is upregulated. Transfection of cancer cells with LAMC2 resulted in the attenuation of ER stress phenotype, accompanied by elevation in mitochondrial membrane potential as well as reduction in reactive oxygen species (ROS) levels and apoptosis. In addition, LAMC2 forms protein complexes with MYH9 and MYH10 to promote mitochondrial aggregation and increased ER-mitochondria interaction at the perinuclear region. Moreover, overexpression of LAMC2 counteracts the effects of ER stress and promotes tumor growth in vivo. Taken together, our results revealed that in complex with MYH9 and MYH10, LAMC2 is essential for promoting ER-mitochondria interaction to alleviate ER stress and allow cancer cells to adapt and proliferate under stressful conditions. This study provides new insights and highlights the promising potential of LAMC2 as a therapeutic target for cancer treatment.

## Introduction

Cancer is a major global public health problem, and remains the leading cause of death for all income levels around the world. In recent years, in addition to routine routes of cancer treatment including surgery, chemotherapy, and radiation, molecular targeted drugs and immune-monitoring drugs have become important areas of research. Despite declining rates of cancer mortality, the challenge of acquired drug resistance may attenuate future progress. The mechanism of tumor drug resistance is complex, involving tumor genetic heterogeneity, epigenetic regulatory changes, endoplasmic reticulum stress (ERS), non-coding RNA regulation, and abnormal expression of drug resistance genes. Among them, ERS and its related pathways are one of the important mechanisms of tumor drug resistance.

Endoplasmic reticulum (ER) stress occurs in response to misfolded/unfolded protein aggregation and calcium homeostasis dysregulation [1]. Various downstream adaptive response mechanisms are activated due to ERS, including unfolded protein response, ER overload response, and caspase-12-mediated cell apoptosis. These response pathways can in turn promote the expression of ER chaperones such as GRP78 and GRP94, thereby generate protective effects, promote adaptation, or induce endogenous apoptosis in stressed cells [2]. ER stress can occur due to unfavorable conditions such as hypoxia, diminished nutrient supply, free radical accumulation, pH drop, cytotoxic drug exposure, radiation, and extracellular microenvironment changes. In eukaryotic cells, there are three main ER stress-related signaling pathways: inositol requires enzyme 1α (IRE1α), PKR-like endoplasmic reticulum kinase (PERK), and activation transcription factor 6α (ATF6α) [3]. Elevated levels of GRP78 are characterized by low levels of chronic ERS, enabling tumor cells to activate adaptive response, which is conducive to the survival of tumor cells under adverse conditions such as chemotherapy or radiotherapy. Based on the fact that most tumor cells have low-level chronic ERS signatures, but normal cells do not, searching for new genes responsible for maintaining low-level chronic ERS signatures and exploring potential targeted intervention will provide novel insights for cancer therapy.

Early studies have shown that Laminin-5γ-2 (LAMC2), an extracellular matrix protein, is involved in cell adhesion, differentiation, migration, signal transduction, neurite growth, and metastasis [4]. Recent research has shown that LAMC2 is highly expressed in lung cancer, undifferentiated thyroid carcinoma, cholangiocarcinoma, laryngeal cancer, ovarian cancer, and pancreatic cancer [5–10]. LAMC2 protein levels have been found elevated in the serum of patients with liver cancer, non-small cell lung cancer (NSCLC), pancreatic cancer, etc [11–13]. Mechanistically, LAMC2 has been shown to promote the progression of pancreatic ductal carcinoma through the EGFR/ERK1/2/AKT/mTOR cascade pathway. In lung cancer, as the downstream target of miR-622/197, LAMC2 was shown to promote cancer cell invasion via the EGFR/ERK1/2-MMP7 pathway [14]. LAMC2 can also modulate tumor microenvironment by regulating macrophage infiltration and extracellular matrix remodeling in NSCLC [15]. In addition, LAMC2 can interact with integrin β1 to promote colorectal cancer tumor budding by activating Yes-related protein [16]. Moreover, studies have indicated that LAMC2 is involved in acquired drug resistance, including platinum and gemicitabine resistance for ovarian and pancreatic cancer patients, respectively.

While the underlying mechanisms of LAMC-induced drug resistance is elusive, investigating regulatory roles of LAMC2 in the cytoplasm may provide new insight. Our study reports that LAMC2 is mainly localized in the endoplasmic reticulum of lung, breast, and liver cancer cells. ER stress-induced the elevation of LAMC2 protein levels, accompanied by increased expression of stress-related proteins PERK, ATF6, CHOP, and GRP78. Moreover, we found that LAMC2 formed protein complexes with myosin heavy-chain 9 (MYH9) and myosin heavy-chain 10 (MYH10), thereby promoting mitochondrial aggregation and ER-mitochondria interaction at the perinuclear region. Silencing LAMC2 reduced mitochondrial membrane potential, increased reactive oxygen species (ROS) levels, and promoted cellular apoptosis. In budding yeast, the maintenance and isolation of mitochondrial DNA relies on the actin cytoskeleton. Defects in the MYH10 gene can lead to a 60% reduction in mitochondrial DNA copy number in mouse embryonic fibroblasts [17]. MYH9 can bind to short transmembrane protein 1 (STMP1) and activate dynamin-related protein 1 (DRP1), which in turn promotes mitochondrial fission and cell migration. The above studies suggest that MYH9 and MYH10 are involved in mitochondrial homeostasis [18]. Mitochondria play an important role in regulating early ER stress. Endoplasmic reticulum and mitochondria are coupled through mitochondria-associated endoplasmic reticulum membranes (MAMs), which mediate oxidative stress, ER stress, Ca^2+^ transport, autophagy, mitochondrial fusion and fission, and apoptosis [19]. Existing studies have shown that ER stress promotes perinuclear mitochondrial aggregation, which results in enhanced ER-mitochondria contact. Increased Ca^2+^ flow from ER to the mitochondria can then augment ATP production to combat ER stress [20]. However, the specific regulatory mechanism of how ER stress promotes ER-mitochondria interaction is unclear.

Taken together, this study proposes that in mitigating ER stress in cancer cells, LAMC2 exerts an emergency stress-resistance response by binding to MYH9 and MYH10 to promote ER-mitochondria interaction around the nucleus. An in-depth investigation of the function and molecular mechanisms of LAMC2 in response to ER stress will provide new insights for cancer progression and targeted intervention.

## Results

### Silencing LAMC2 promotes ER stress, apoptosis, and mitochondrial dysfunction

The oncogenic capacity of LAMC2 has been well-documented in lung cancer, undifferentiated thyroid carcinoma, cholangiocarcinoma, laryngeal cancer, ovarian cancer and pancreatic cancer. We began our study by examining the correlation between LAMC2 and pan-cancer using The Cancer Genome Atlas (TCGA) database. As our results indicated, of 33 different cancer types, LAMC2 is overexpressed in 21, reduced in 7, and unassociated with five cancer types (Supp. Fig. 1A). In addition, we examined the correlation between LAMC2 and overall survival for each cancer type. Results showed that in LAMC2 overexpressing tumors, LAMC2 is negatively correlated with overall survival (Supp. Fig. 1B). In addition, Progression Free Interval (PFI, Supp. Fig. 1C) and Disease Specific Survival (DSS, Supp. Fig. 1D) analyses showed similar trends. These results suggested that LAMC2 is positively associated with tumor progression and poor prognosis.

**Fig. 1:**
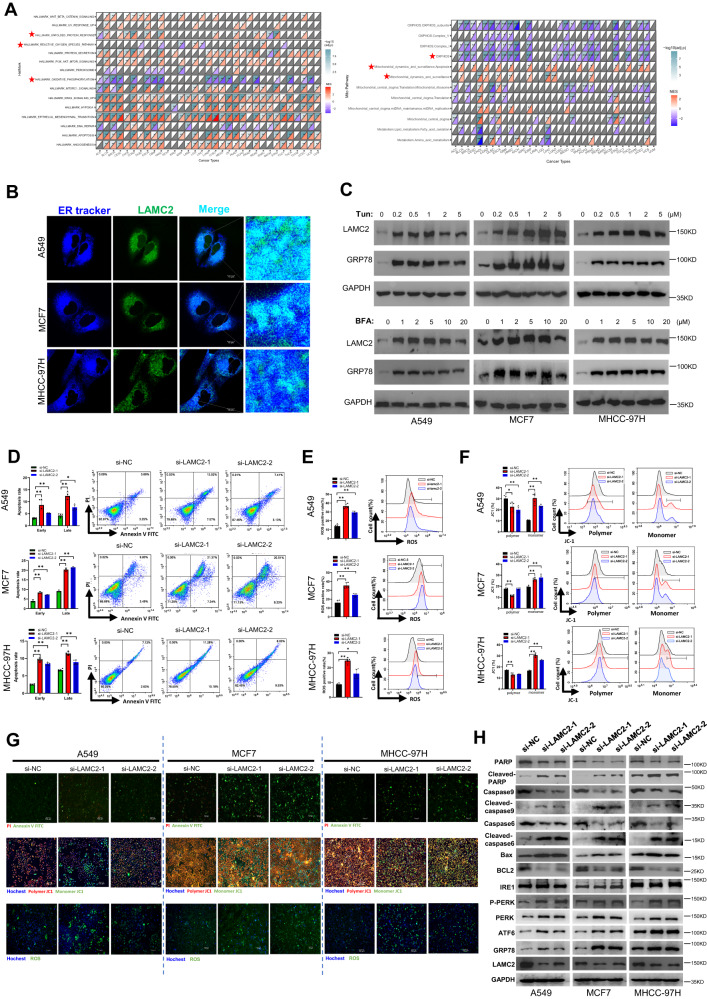
Silencing LAMC2 promotes ER stress, apoptosis, and mitochondrial dysfunction. **A** TCGA pan-cancer database was used to analyze hallmarks (left) and mitochondria-related pathways (right) significantly correlated with LAMC2 expression. **B** Live-cell fluorescence images of co-localization between LAMC2 (green) and ER (blue) in A549, MCF7, and MHCC-97H cells. **C** Western blots of LAMC2, GRP78, and GAPDH in A549, MCF7, and MHCC-97H cells treated with Tun or BFA. **D** Annexin V/FITC flow cytometry analysis of early and late cellular apoptosis in LAMC2 knockdown cancer cells. **E** Flow cytometry analysis of ROS production in LAMC2 knockdown cancer cells. **F** Flow cytometry analysis of mitochondrial membrane potential in LAMC2 knockdown cells represented by polymer and monomer levels. **G** Live-cell fluorescence imaging was used to detect Annexin V/FITC, polymer JC1, monomer JC1, and ROS in A549, MCF7, and MHCC-97H cells. **H** Western blots of PARP, cleaved-PARP, caspase 9, cleaved caspase 9, caspase 6, cleaved caspase 6, Bax, BCL2, IRE1, p-PERK, PERK, ATF6, GRP78, LAMC2, and GAPDH protein levels. Graphical data were presented as mean± SEM. *p < 0.05, **p < 0.01.

In addition, pan-cancer Gene Set Enrichment Analysis (GSEA) analysis was also carried out using the TCGA database for the same 33 cancer types. LAMC2 was found significantly correlated with unfolded protein response, which is a crucial ER stress adaptive response mechanism, as well as physiological processes including reactive oxygen species (ROS) generation and oxidative phosphorylation (Fig. 1A left). ROS production and oxidative phosphorylation are important biological processes related to the mitochondria. Therefore, we performed another GSEA analysis to examine the potential correlation between LAMC2 and mitochondria-specific signaling pathways. Results indicated that LAMC2 was negatively correlated with oxidative phosphorylation pathways, and positively correlated with mitochondrial dynamics and surveillance, as well as apoptosis (Fig. 1A, right). These data indicated that LAMC2 is involved in ER stress- and mitochondrial-related biological processes. To verify the function of LAMC2 in ER stress response, we first confirmed subcellular localization of LAMC2 in the ER using live-cell fluorescence detection. As shown in Fig. 1B, LAMC2 can be found to localize in the ER of A549, MCF7, and MHCC-97H cells. In addition, tunicamycin (Tun) and Bredfeldin A (BFA) treatment, which were used to induce ER stress, significantly promoted LAMC2 and GRP78 (ER stress marker) protein levels (Fig. 1C). Our data also showed that GRP78 and LAMC2 protein levels increased with increasing treatment time (Supp. Fig. 2D). Moreover, mRNA levels of LAMC2 were also increased post tunicamycin treatment (Supp. Fig. 2B). To explore whether LAMC2 is involved in ER stress-induced ROS production, mitochondrial dysfunction, and consequent apoptosis, we inhibited LAMC2 expression in A549, MCF7, and MHCC-97H cells using siRNA. Flow cytometry results indicated that LAMC2 silencing significantly induced early and late apoptosis (Fig. 1D), as well as ROS production (Fig. 1E). Moreover, while the proportion of JCI polymer cells decreased, monomer cells increased in si-LAMC2 cells, suggesting that silencing LAMC2 led to a reduction in mitochondrial membrane potential (Fig. 1F). Live-cell fluorescence staining further confirmed these results (Fig. 1G and Supp. Fig. 3A–C). Additionally, western blotting analyses showed that LAMC2 knockdown led to increased protein levels of Cleaved-PARP, Cleaved-caspase9, Cleaved-caspase6, and BAX, which are known to promote apoptosis. On the other hand, BCL2 protein levels were reduced. In addition, ER stress proteins GRP78, ATF6, PERK, and phosphorylated PERK were upregulated in LAMC2 knockdown cells. Interestingly, IRE1 protein level was unaffected (Fig. 1H). The above results demonstrated that ER stress induced abnormal elevation of LAMC2 protein levels. Repression of LAMC2, however, elicited ER stress and impaired mitochondrial function, ultimately triggering apoptosis in cancer cells. Collectively, our data suggested that under ER stress, LAMC2 protein level is upregulated to enhance mitochondrial function and mitigate stress-induced cell death.

### LAMC2 mitigates ER stress and is associated with ER-mitochondria interaction

**Fig. 2:**
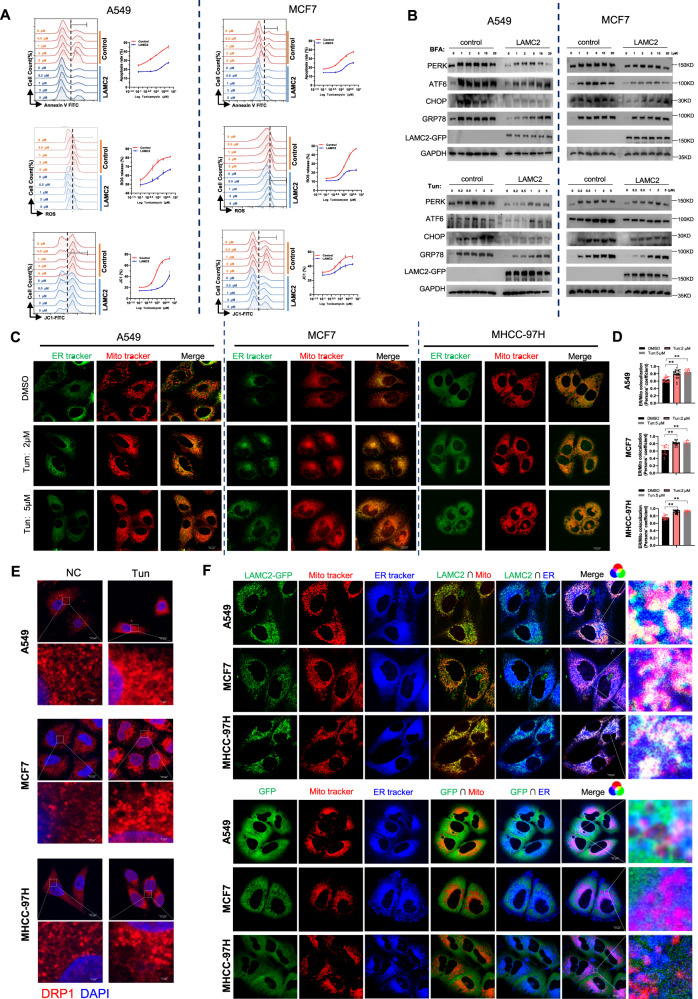
LAMC2 mitigates ER stress and is associated with ER-mitochondria interaction. **A** Flow cytometry analyses of Annexin V/FITC, ROS, and JC1-FITC in control and LAMC2 overexpressing A549 and MCF7 cells treated with Tun. **B** Western blots of PERK, ATF6, CHOP, GRP78, LAMC2-GFP, and GAPDH protein levels in control and LAMC2 overexpressing A549 and MCF7 cells treated with either BFA or Tun. **C** Live-cell fluorescence images of co-localization between ER (green) and mitochondria (red) in A549, MCF7, and MHCC-97H cells treated with DMSO or Tun. **D** Quantification of ER-mitochondria co-localization. **E** Immunofluorescence images of DRP1(red) and DAPI (blue) in A549, MCF7, and MHCC-97H cells. **F** Live-cell fluorescence images of LAMC2-GFP (green), mitochondria (red), and ER (blue), as well as co-localization between LAMC2/mitochondria, LAMC2/ER, and LAMC2/mitochondria/ER in A549, MCF7, and MHCC-97H cells. GFP was used as control. Graphical data were presented as mean ± SEM. *p < 0.05, **p < 0.01.

To confirm the role of LAMC2 in ER stress response, we treated LAMC2 overexpressing A549 and MCF7 cells with tunicamycin and evaluated the effect of ER stress on apoptosis, ROS production, and mitochondrial membrane potential. As shown in Fig. 2A, Tun-induced apoptosis, ROS production, and reduction in membrane potential were significantly lower in the LAMC2 overexpressing cells compared to the control cells. Moreover, BFA- and Tun-induced increase in ER stress makers such as PERK, ATF6, CHOP, and GRP78 were also significantly less in the LAMC2 overexpressing group (Fig. 2B). These results suggested that LAMC2 overexpression can reverse ER stress-induced cell apoptosis, as well as increase ROS production and decrease mitochondria membrane potential. Furthermore, treatment of A549 cells with si-LAMC2 significantly enhanced the induction of endoplasmic reticulum stress markers (GRP78, PERK, and ATF6) by Tun compared to the control group. Consistent with previous findings, LAMC2 knockdown resulted in increased cell apoptosis under non-Tun treatment conditions when compared to the control group. Moreover, under Tun treatment conditions, the extent of cell apoptosis was further augmented in the LAMC2 knockdown group relative to the control group (Supp. Fig. 4C, D). Having validated the impact of LAMC2 on both ER stress and mitochondria-related biological processes, we next examined potential changes in the physiological interaction between ER and mitochondria under ER stress using live-cell fluorescent staining. Results indicated that compared to the control group, Tun treated cells showed significantly higher ER-mitochondria interaction (Fig. 2C, D). To corroborate these results, we showed that Tun treatment also promoted dynamin-related protein 1 (DRP1) deposition near the nucleus (Fig. 2E). DRP1 is an important player in regulating mitochondria dynamics, and has been shown to facilitate ER and mitochondria interaction [21]. Therefore, our results validated that Tun-induced ER stress promoted ER-mitochondria interaction at the perinuclear region. Noted, increase in ER and mitochondria interaction has been previously reported as a potential mechanism for relieving ER stress [22–25].

In effort to determine whether LAMC2 participates in enhancing ER-mitochondria interaction, we transfected GFP-tagged LAMC2 vectors into A549, MCF7, and MHCC-97H cells, and examined the co-localization between LAMC2, ER, and mitochondria using live-cell fluorescence microscopy. Imaging results showed that LAMC2 co-localized strongly with ER and mitochondria at the ER-mitochondria junction, which is also where DRP1 can be found (Fig. 2F). In contrast, GFP in the control group localized in a diffused and non-specific manner. Overall, our data demonstrated that LAMC2 not only participates in preventing ER stress-induced apoptosis and mitochondria dysfunction, but can also physically interact with ER and mitochondria at the ER-mitochondria junction. These results suggested that LAMC2 may mitigate ER stress at least in part via promoting ER-mitochondria interaction.

### LAMC2 interacts with MYH9 and MYH10

**Fig. 3:**
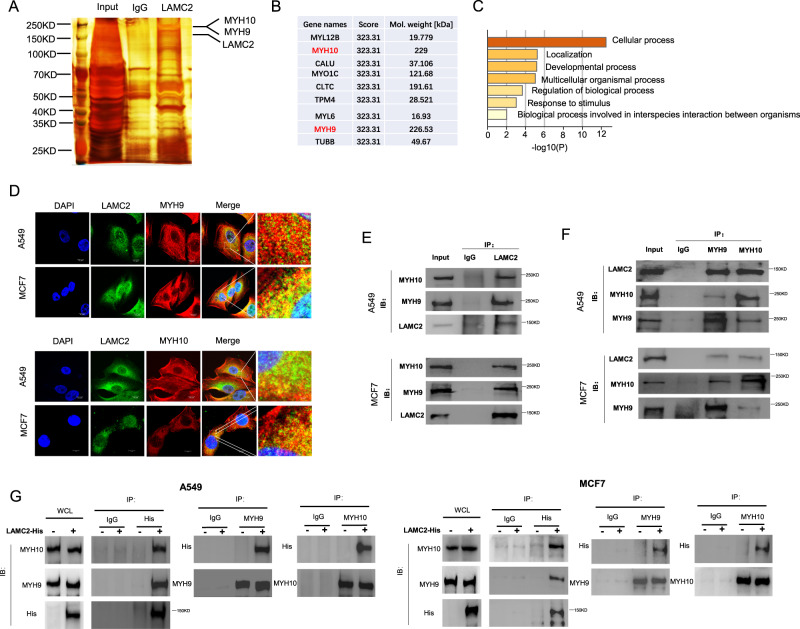
LAMC2 interacts with MYH9 and MYH10. **A** Silver staining image of proteins co-purified with LAMC2. **B** Table showing top proteins with high affinity for LAMC2. **C** KEGG pathway analysis of biological processes associated with top 20 proteins with highest binding affinity to LAMC2. **D** Immunofluorescence images of DAPI (blue), LAMC2 (green), and MYH9/MYH10 (red), as well as co-localization between LAMC2 and MYH9, LAMC2 and MYH10 in A549 and MCF7 cells. **E** Co-immunoprecipitation of LAMC2 with MYH9 and MYH10 in A549 and MCF7 cells. **F** Co-immunoprecipitation of MYH9 and MYH10 with LAMC2 in A549 and MCF7 cells. **G** Co-immunoprecipitation of LAMC2-His with MYH9 and MYH10 in A549 and MCF7 cells.

To further explore the underlying mechanism of LAMC2 in regulating ER- and mitochondria-related biological functions, we first examined the potential binding partners of LAMC2 in A549 cells through immunoprecipitation coupled to mass spectrometry (IP-MS). As shown in Fig. 3A, B, most of the proteins bound by LAMC2 are cytoskeletal proteins. Silver staining results in MCF7 and MHCC-97H cells are shown in Supplementary Fig. 4E. KEGG pathway analysis showed that the main biological processes associated with the top 20 proteins with highest binding affinity to LAMC2 include localization, response to stimulus, and organelle interaction (Fig. 3C). Likewise, GO and protein-protein interaction (PPI) analyses revealed that LAMC2-bound proteins are highly associated with ER processing and multi-organism metabolic processes (Supp. Fig. 4F–H). Collectively, these results confirmed that LAMC2 and its binding partners are highly correlated with ER-related biological processes. Among the top binding proteins, we found MYH9 and MYH10 proteins to be tightly associated with ER and mitochondria dynamics [26]. Thus, we examined the physical interaction between MYH9, MYH10, and LAMC2 through immunofluorescence imaging. In both A549 and MCF7 cells, LAMC2 was found to co-localize with both MYH9 and MYH10 near the nuclear membrane (Fig. 3D). In addition, co-immunoprecipitation (Co-IP) experiments confirmed the ability for LAMC2 to bind both MYH9 and MYH10 (Fig. 3E). Interestingly, in addition to separately interacting with LAMC2, Co-IP results also demonstrated the physical interaction between MYH9 and MYH10 (Fig. 3F). These results suggested that LAMC2, MYH9, and MYH10 proteins can exist in the form of a complex. To further validate the binding relationship between LAMC2, MYH9, and MYH10, we transfected His-tagged LAMC2 plasmid into A549 and MCF7 cells. Co-IP results confirmed the binding interaction between MYH9, MYH10, and exogenous LAMC2 (Fig. 3G). As previously mentioned, MYH9 and MYH10 are important for ER and mitochondrial dynamics. Therefore, binding between LAMC2, MYH9, and MYH10 may be involved in regulating ER-mitochondria interaction.

### ER stress stimulates the formation of LAMC2/MYH9/MYH10 protein complex

**Fig. 4:**
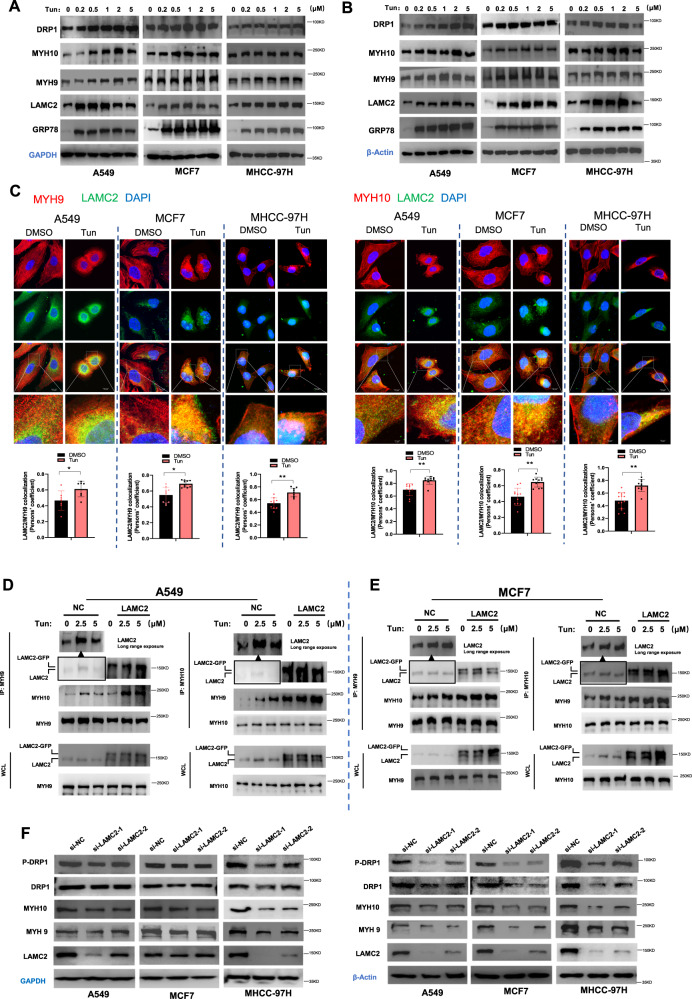
ER stress stimulates the formation of LAMC2/MYH9/MYH10 protein complex. **A** Western blots of DRP1, MYH10, MYH9, LAMC2, and GRP78 with GAPDH as internal reference for A549, MCF7, and MHCC-97H cells treated with Tun. **B** Western blots of DRP1, MYH10, MYH9, LAMC2, and GRP78 with β-actin as internal reference for A549, MCF7, and MHCC-97H cells treated with Tun. **C** Immunofluorescence images of DAPI (blue), LAMC2 (green), MYH9/MYH10 (red), as well as co-localization between LAMC2 and MYH9, LAMC2 and MYH10 in A549, MCF7, and MHCC-97H cells treated with either DMSO or Tun. Quantification graphs demonstrate co-localization between LAMC2/MYH9 and LAMC2/MYH10 (bottom). **D** Co-immunoprecipitation of LAMC2 with MYH9 and MYH10 in control and LAMC2 overexpressing A549 cells treated with Tun. **E** Co-immunoprecipitation of LAMC2 with MYH9 and MYH10 in control and LAMC2 overexpressing MCF7 cells treated with Tun. **F** Western blots of P-DRP1, DRP1, MYH10, MYH9, LAMC2, and GAPDH in control and LAMC2 knockdown cancer cells (left). Western blots of P-DRP1, DRP1, MYH10, MYH9, LAMC2, and β-actin protein levels in control and LAMC2 knockdown cancer cells (right). Graphical data were presented as mean± SEM. *p < 0.05, **p < 0.01.

To examine potential changes in MYH9 and MYH10 under ER stress, we treated A549, MCF7, and MHCC-97H cells with various concentrations of tunicamycin, and found that protein levels of MYH9, MYH10, LAMC2, GRP78, as well as DRP1 significantly increased (Fig. 4A, B). In addition, qRT-PCR results showed that MYH9 and MYH10 mRNA levels were also slightly increased (Supp. Fig. 2B). Considering that GAPDH is associated with metabolism, its use is suitable for quantitative analysis of cytoskeletal proteins. However, since DRP1 is a key protein associated with mitochondria metabolism, we chose to repeat the western blotting analyses using β-actin as the internal reference (Fig. 4B). As mentioned earlier, ER-mitochondria localization at the perinuclear region is an indication of ER stress response. Corroborating this phenomenon, our immunofluorescence imaging results indicated that tunicamycin treatment significantly enhanced the co-localization between LAMC2, MYH9, and MYH10 near the nuclear membrane (Fig. 4C). Quantification of the fluorescent images confirmed this finding. In addition, Co-IP experiments showed that the increased binding of MYH9 to LAMC2 and MYH10 after tunicamycin treatment was more evident in the LAMC2 overexpressing A549 cells compared to the control cells (Fig. 4D, left). Similarly, binding of MYH10 to LAMC2 and MYH9 after tunicamycin treatment was more significant in the LAMC2 overexpressing cells (Fig. 4D, right). Consistent results were also found in MCF7 cells (Fig. 4E). These results suggested that ER stress enhanced protein binding between LAMC2, MYH9, and MYH10. To examine the effect of LAMC2 on MYH9 and MYH10, we performed western blotting analyses using si-LAMC2 cancer cells, and showed that silencing LAMC2 significantly reduced the protein levels of MYH9, MYH10, as well as DRP1 and phosphorylated DRP1 (Fig. 4F). However, qRT-PCR results demonstrated that mRNA levels of MYH9 and MYH10 were not changed in si-LAMC2 cells (Supp. Fig. 2A), which suggests that the interaction between LAMC2, MYH9, and MYH10 is mainly at the protein level. Collectively, our data demonstrated that ER stress enhances LAMC2 expression and promotes LAMC2/MYH9/MYH10 protein complex formation near the nuclear membrane, which may be the underlying mechanism for facilitating ER-mitochondria interaction and alleviating ER stress-induced apoptosis.

### LAMC2 overexpression promotes ER-mitochondria interaction

**Fig. 5:**
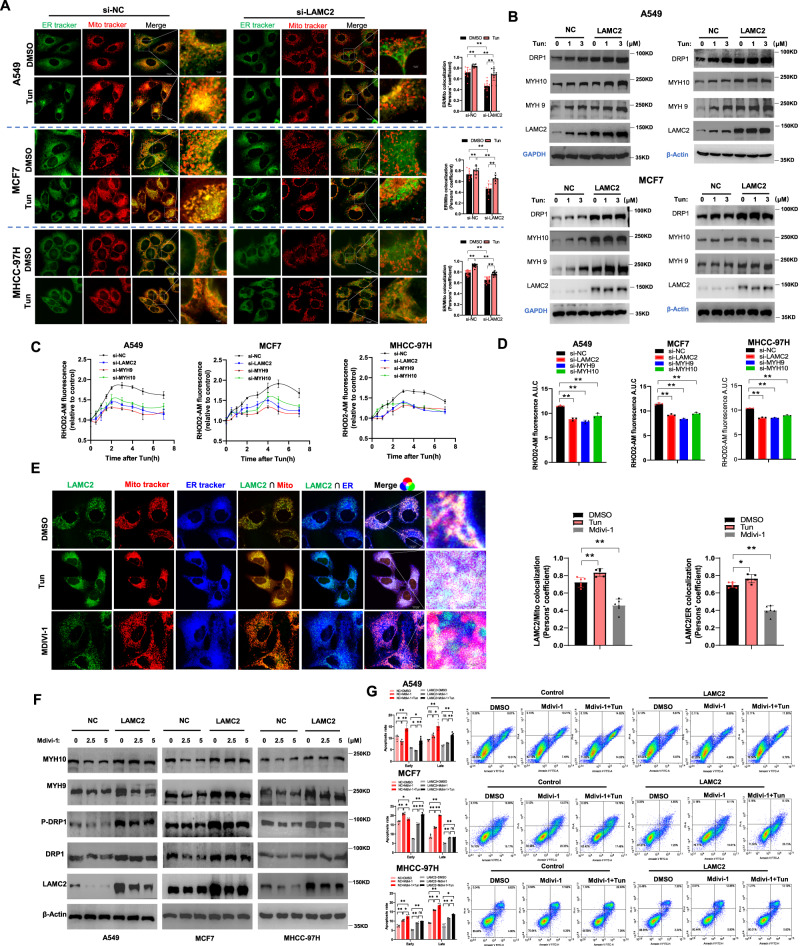
LAMC2 overexpresison promotes ER-mitochondria interaction. **A** Live-cell fluorescence images of co-localization between ER (green) and mitochondria (red) in control and LAMC2 knockdown cancer cells treated with either DMSO or Tun. Quantification of ER-mitochondria co-localization (right). **B** Western blots of DPR1, MYH10, MYH9, LAMC2, GAPDH, and β-actin protein levels in control and LAMC2 overexpressing A549 and MCF7 cells treated with Tun. **C** Mitochondrial calcium levels detected by Rhod-2-AM Cell Permeable Kit in control and LAMC2 knockdown cancer cells. **D** Graphical representation of Rhod-2-AM fluorescence A.U.C in control and LAMC2 knockdown cancer cells. **E** Live-cell fluorescence images of LAMC2 (green), mitochondria (red), and ER (blue), as well as co-localization between LAMC2/mitochondria, LAMC2/ER, and LAMC2/mitochondria/ER in A549, MCF7, and MHCC-97H cells treated with DMSO, Tun, or MDIVI-1. Quantification of LAMC2/Mitochondria and LAMC2/ER co-localization (right). **F** Western blots of MYH10, MYH9, P-DRP1, DRP1, LAMC2, and β-actin protein levels in control and LAMC2 overexpressing cancer cells treated with MDIVI-1. **G** Flow cytometry analysis of early and late cellular apoptosis in control and LAMC2 overexpressing cancer cells treated with DMSO, MDIVI-1, or Mdivi-1+Tun. Graphical data were presented as mean± SEM. *p < 0.05, **p < 0.01.

As previously shown in Fig. 2C, tunicamycin treatment promoted ER-mitochondria co-localization. To establish that this phenomenon is driven by LAMC2 overexpression, we used live-cell fluorescent imaging to examine ER-mitochondria co-localization in LAMC2 silencing cells. As shown in Fig. 5A, while tunicamycin treatment enhanced ER-mitochondria co-localization, the degree of co-localization was significantly lower in the si-LAMC2 cells. This result suggested that LAMC2 expression is important for promoting ER-mitochondria interaction at the perinuclear region. In addition, we showed that LAMC2 overexpression and tunicamycin treatment synergistically promoted protein levels of DRP1, MYH9, and MYH10 in A549 and MCF7 cells (Fig. 5B). Using RHOD2-AM fluorescent staining, we also showed that silencing LAMC2, MYH9 or MYH10 inhibited mitochondria calcium levels (Fig. 5C, D). Furthermore, we detected the degree of co-localization between LAMC2, mitochondria, and ER under tunicamycin and Mdivi-1 treatment. As a mitochondrial-division inhibitor, Mdivi-1 can be used to inhibit DRP1 levels, which would subsequently hinder ER-mitochondria interaction [21, 27]. As shown in Fig. 5E, while tunicamycin treatment promoted LAMC2 co-localization with the mitochondria and ER, Mdivi-1 reduced binding between the three. Moreover, while Mdivi-1 treatment reduced the protein levels of MYH9, MYH10, P-DRP1, and DRP1, overexpression of LAMC2 reversed this effect (Fig. 5F). Lastly, we treated control and LAMC2 overexpressing cells with either Mdivi-1 or Mdivi-1+Tun to examine potential changes in cell apoptosis via flow cytometry. As demonstrated in Fig. 5G, while Mdivi-1 alone can promote early and late apoptosis, the effect was significantly more evident in the combined treatment (MDIVI-1+Tun) group. Compared to the control group, LAMC2 overexpression was able to reverse Mdivi-1 and Tun-induced cell apoptosis. Overall, our data suggested that upon ER stress, LAMC2 expression is elevated in part to promote ER-mitochondria interaction.

### MYH9 and MYH10 regulates ER stress and mitochondrial function

**Fig. 6:**
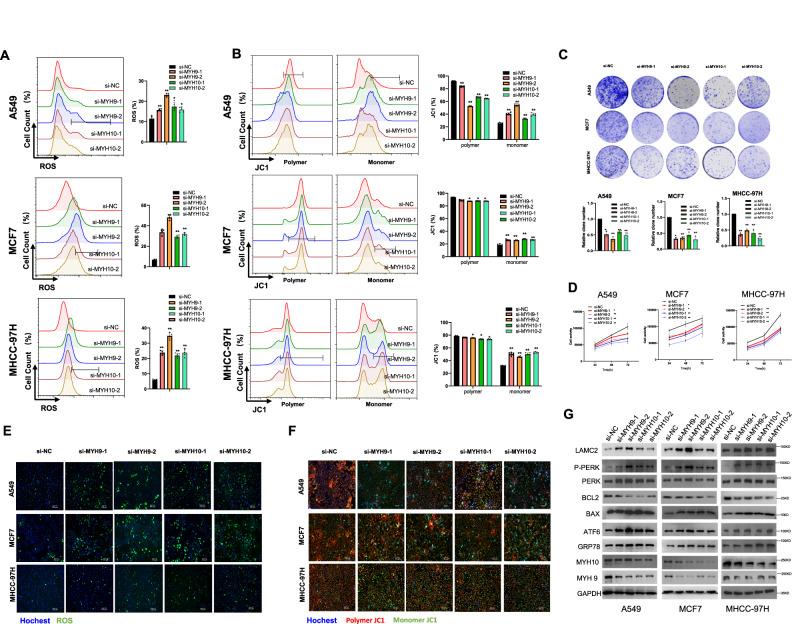
MYH9 and MYH10 regulates ER stress and mitochondrial function. **A** Flow cytometry analysis of ROS production in MYH9 and MYH10 knockdown cancer cells. **B** Flow cytometry analysis of mitochondrial membrane potential in MYH9 and MYH10 knockdown cells represented by polymer and monomer levels. **C** Colony formation of MYH9 and MYH10 knockdown cancer cells. Quantification of clone number (bottom). **D** Cell activity levels of MYH9 and MYH10 knockdown cancer cells. **E** Live-cell fluorescence imaging was used to ROS in MYH9 and MYH10 knockdown cancer cells. **F** Live-cell fluorescence imaging was used to polymer JC1 and monomer JC1 in MYH9 and MYH10 knockdown cancer cells. **G** Western blots of LAMC2, P-PERK, PERK, BCL2, BAX, ATF6, GRP78, MYH10, MYH9, and GAPDH in MYH9 and MYH10 knockdown cancer cells. Graphical data were presented as mean ± SEM. *p < 0.05, **p < 0.01.

While previous studies have indicated that MYH9 and MYH10 are important for mitochondria dynamics, and may play a role in ER stress, it is unclear whether they participate in similar cellular physiological processes as LAMC2, including ROS production and mitochondrial membrane potential maintenance. Through flow cytometry analyses, we measured ROS production and mitochondrial membrane potential in MYH9 and MYH10 knockdown cells. As shown in Fig. 6A, B, silencing MYH9 and MYH10 significantly promoted ROS production, and reduced mitochondrial membrane potential. In addition, we showed that MYH9 and MYH10 knockdown cancer cells exhibited lower colony formation and cell viability compared to the control group cells (Fig. 6C, D). Live-cell fluorescent imaging confirmed the increase in ROS production and decrease in mitochondrial membrane potential in MYH9 and MYH10 knockdown cells (Fig. 6E, F and Supp. Fig. 3D, E). Lastly, through western blotting analyses, we showed that silencing MYH9 and MYH10 promoted ER stress markers such as GRP78, ATF6, PERK, and P-PERK (Fig. 6G). On the other hand, BCL2 was reduced while BAX was elevated in MYH9 and MYH10 knockdown cells, indicating reduced cell apoptosis. In addition, knockdown of MYH9 promoted LAMC2 but inhibited MYH10 protein levels. Similarly, knockdown of MYH10 promoted LAMC2 but reduced MYH9 protein levels. The increase in LAMC2 protein may be a result of si-MYH9- or si-MYH10-induced ER stress. At the mRNA level, knockdown of MYH9 in A549 and MHCC-97H cells promoted LAMC2 mRNA levels but MYH10 mRNA levels remained unchanged. Similarly, knockdown of MYH10 elevated LAMC2 mRNA levels, while MYH9 mRNA levels remained unchanged (Supp. Fig. 2C). However, in MCF7 cells, knockdown of MYH9 promoted the mRNA levels of both LAMC2 and MYH10. Same were found for MYH10 knockdown cells (Supp. Fig. 2C). The varied qRT-PCR results further suggested that the interaction between LAMC2, MYH9, and MYH10 are mainly at the protein level. Overall, data from this section confirmed that LAMC2, MYH9, and MYH10 exist in a complex, and that knockdown of any one of the proteins will affect the protein levels of the other two.

### LAMC2 enhances ER-mitochondria interaction and mitigates ER stress through binding to MYH9 and MYH10

**Fig. 7:**
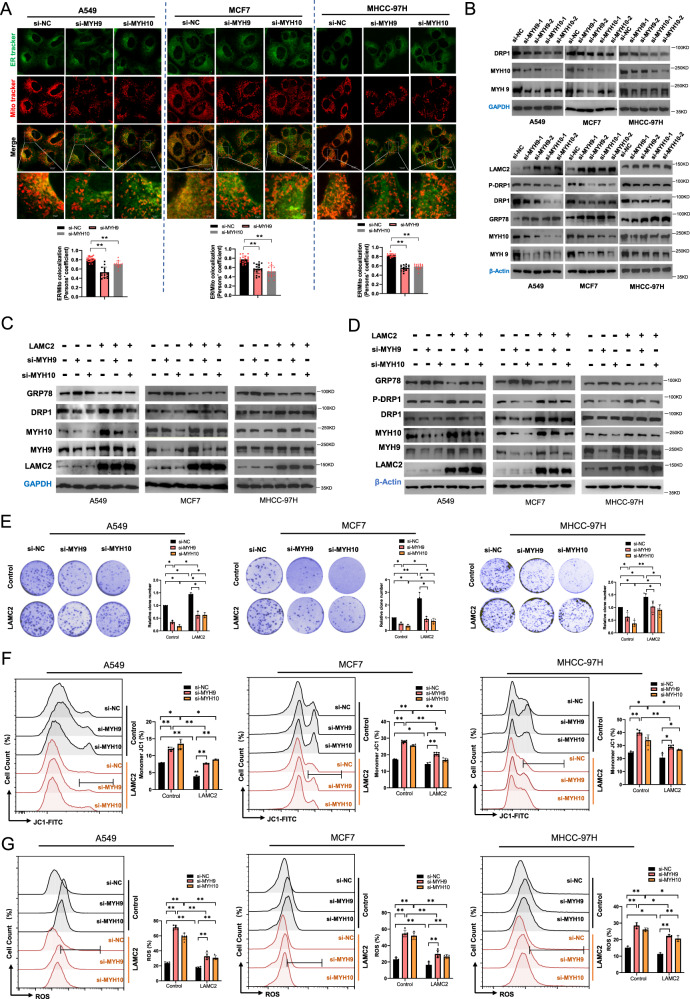
LAMC2 enhances ER-mitochondria interaction and mitigates ER stress through binding to MYH9 and MYH10. **A** Live-cell fluorescence images of co-localization between ER (green) and mitochondria (red) in MYH9 and MYH10 knockdown cancer cells. Quantification of ER/mitochondria co-localization (bottom). **B** Western blots of DRP1, MYH10, MYH9, GAPDH, and β-actin protein levels in MYH9 and MYH10 knockdown cancer cells. **C** Western blots of GRP78, DRP1, MYH10, MYH9, LAMC2, and GAPDH in co-transfected cancer cells. Cells were co-transfected with control, si-MYH9, si-MYH10, LAMC2, LAMC2+si-MYH9, or LAMC2+si-MYH10. **D** Western blots of GRP78, DRP1, MYH10, MYH9, LAMC2, and β-actin in co-transfected cancer cells. Cells were co-transfected with control, si-MYH9, si-MYH10, LAMC2, LAMC2+si-MYH9, or LAMC2+si-MYH10. **E** Colony formation of control and LAMC2 overexpressing cancer cells co-transfected with either si-MYH9 or si-MYH10. Relative clone number (right). **F** Flow cytometry analysis of JC1 (monomer %) in control and LAMC2 overexpressing cancer cells co-transfected with either si-MYH9 or si-MYH10 cancer cells. **G** Flow cytometry analysis of ROS levels in control and LAMC2 overexpressing cancer cells co-transfected with either si-MYH9 or si-MYH10 cancer cells. Graphical data were presented as mean ± SEM. *p < 0.05, **p < 0.01.

To establish the effect of MYH9 and MYH10 on ER-mitochondria interaction, we knocked down either MYH9 or MYH10 in A549, MCF7, and MHCC-97H cells. Using live-cell fluorescence imaging, we showed that silencing either MYH9 or MYH10 strongly reduced ER and mitochondria co-localization (Fig. 7A). Moreover, the amount of fluorescence for mitochondria was significantly reduced in MYH9 and MYH10 knockdown cells. These results are consistent with that of LAMC2 knockdown cells (Fig. 5A). Western blotting analyses showed that knockdown of either MYH9 or MYH10 strongly induced GRP78 protein elevation, but reduced DRP1 and phosphorylated DRP1 protein levels (Fig. 7B), indicating that lack of MYH9 or MYH10 promoted ER stress and reduced ER-mitochondria interaction. Noted, knockdown of MYH9 reduced protein levels of MYH10, and vice versa. In addition, we silenced either MYH9 or MYH10 along with overexpression of LAMC2 to examine potential rescuing effects. Western blotting results showed that LAMC2 overexpression could partially offset the effects of si-MYH9 and si-MYH10 on GRP78 and DRP1 protein levels (Fig. 7C, D). These results further demonstrate that LAMC2 regulates ER-mitochondria interaction through MYH9 and MYH10. Furthermore, we showed that while LAMC2 overexpression promoted colony formation, knockdown of either MYH9 or MYH10 could counteract the oncogenic capacity of LAMC2 (Fig. 7E). Lastly, flow cytometry analyses showed that silencing either MYH9 or MYH10 effectively prevented LAMC2-induced increase in mitochondrial membrane potential (Fig. 7F), as well as decrease in ROS production (Fig. 7G). These results suggested that the lack of MYH9 or MYH10 prevents the ability for LAMC2 to inhibit ROS production and bolster mitochondrial function. Collectively, our data suggested that LAMC2 mitigates ER stress by promoting ER-mitochondria interaction by acting in complex with MYH9 and MYH10.

### LAMC2 counteracts the effect of ER stress and promotes tumor growth in vivo

**Fig. 8:**
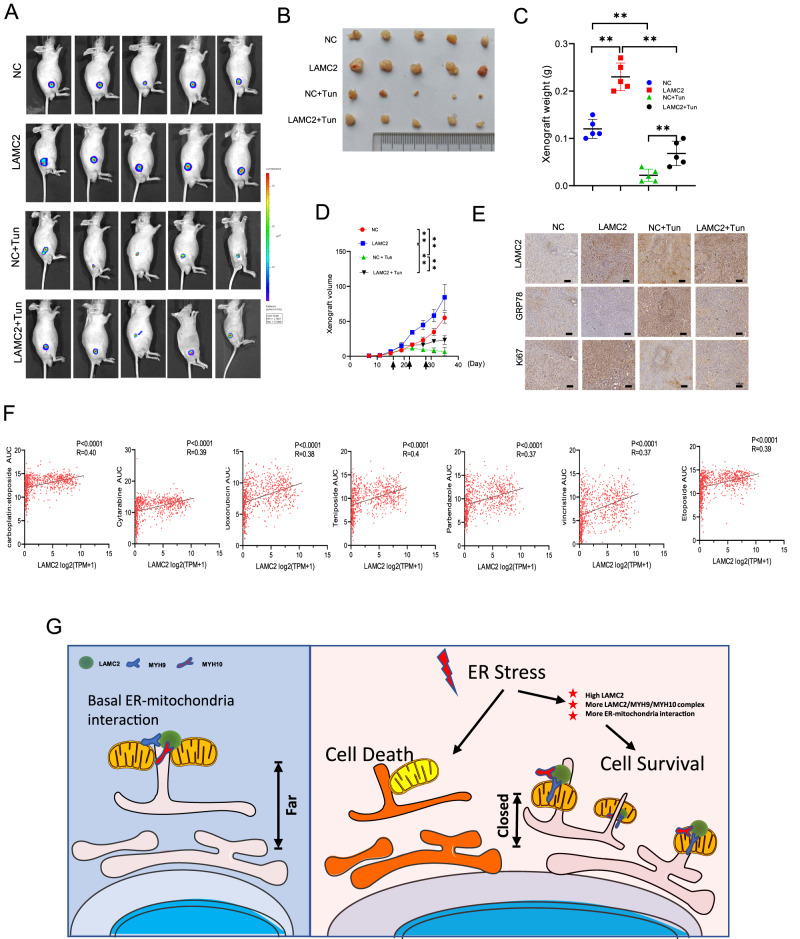
LAMC2 counteracts the effect of ER stress and promotes tumor growth in vivo. **A** Bioluminescence imaging of xenograft at day 35 in BALB/c-nude mice injected with either control, LAMC2, control+Tun, or LAMC2+Tun A549 cells. **B** Image of extracted tumor tissue, demonstrating gross morphology and size. **C** Quantification of xenograft weight. **D** Quantification of xenograft volume. **E** IHC staining of LAMC2, GRP78, and Ki67 in tumor tissues. **F** Correlation between drug sensitivity and LAMC2 expression, analyzed using CCLE and AUC drug sensitivity data based on seven chemotherapeutic drugs downloaded from PRISM. **G** Graphical representation of increased ER-mitochondria interaction near the nucleus in response to ER stress and elevated LAMC2 levels. *p < 0.05, **p < 0.01.

Prolonged ER stress and subsequent cellular apoptosis are detrimental for tumor growth. To examine the ability for LAMC2 to combat ER stress and promote tumor growth in vivo, we analyzed the real-time tumor growth in nude mice injected with either control or LAMC2 plasmid A549 cells treated with tunicamycin. As shown in Fig. 8A–D, xenografts in the LAMC2 group were significantly bigger than that of the control group. Moreover, while xenograft size, weight, and volume were significantly reduced through tunicamycin treatment in the control group, the effects were less significant in the LAMC2 group. In other words, LAMC2 overexpression efficiently impeded the tumor-inhibiting effect of tunicamycin. Immunohistochemistry staining of the tumor tissues showed that LAMC2 overexpression effectively reduced GRP78, and promoted Ki67 protein levels (Fig. 8E). These results strongly suggested that LAMC2 elicits tumor-promoting capacity by inhibiting ER stress. Interestingly, through bioinformatics analysis using the Cancer cell gene expression (CCLE) and profiling relative inhibition simultaneously in mixtures (PRISM) databases, we found that LAMC2 is positively correlated with seven chemotherapy drugs, including carboplatin, cytarabine, doxorubicin, teniposide, parbendazole, vincristine, and etoposide (Fig. 8F). Overall, as shown in Fig. 8G, results from our study provided compelling evidence that under ER stress, LAMC2 promotes tumor growth by enhancing protein complex formation with MYH9 and MYH10 to facilitate ER-mitochondria interaction, thereby mitigating ER stress-induced cell death.

## Discussion

The capacity for cancer cells to resist cell death via activation of autophagy, ER stress response, drug resistance genes, and related pathways, is a major challenge for cancer therapy. Among the above-mentioned mechanisms, ER stress response plays an important regulatory role in cell death resistance. In addition to classical ER stress proteins such as IRE1α, PERK, ATF6α, GRP78, CHOP and XBP1, new proteins have been discovered to participate in ER stress protection, including GADD34, RRAD, p58IP and SEL1L [28–32]. Through bioinformatic analysis using the TCGA database, we found that LAMC2 was highly expressed in a wide range of tumor tissues, and that GSEA analysis showed LAMC2 was positively correlated with ER stress non-folding protein response, as well as mitochondrial dynamics and apoptosis monitoring, suggesting that LAMC2 may be a new ER stress-related protein. Currently available literature suggests that as an extracellular matrix protein, LAMC2 is involved in the proliferation, invasion, metastasis, and drug resistance in tumors [33, 34]. Elevated LAMC2 expression under ER stress stimulation has been previously confirmed by several studies, but its underlying regulatory mechanism is unclear. We found that LAMC2 is mainly localized to the ER in tumor cells, and under ER stress, both LAMC2 and GRP78 expressions were elevated. In addition, overexpression of LAMC2 inhibited the elevation of GRP78, ATF6, PERK, and CHOP, which are BFA- and Tun-induced ER stress-related proteins. In cancer cells, knockdown LAMC2 promoted apoptosis, while its overexpression inhibited apoptosis, indicating that LAMC2 inhibited the occurrence of Tun-induced apoptosis via alleviating ER stress. Results from animal experiments confirmed the tumor-promoting capacity of LAMC2 under ER stress. Moreover, our data showed that LAMC2 was involved in ER stress-induced mitochondrial dysfunction, which resulted in increased ROS production, and reduced mitochondrial membrane potential. Specifically, knockdown of LAMC2 increased ROS and decreased mitochondrial membrane potential, while overexpression of LAMC2 significantly reduced free radical levels in tumor cells and increased mitochondrial membrane potential. In addition, our data demonstrated that under Tun-induced ER stress, co-localization between ER and mitochondria is increased near the nuclear membrane. Moreover, increased expression of mitochondria dynamic protein DRP1 suggested that the mitochondria and ER may have synergistic effects during ER stress. More important, our results showed that LAMC2 and mitochondria co-localized with ER, suggesting that LAMC2 may be involved in the ER-mitochondria interaction.

The ER and mitochondria are two important organelles that are highly dynamic in eukaryotic cells, and are physically connected to form the mitochondria-associated ER membranes. MAMs participate in a variety of important biological roles including Ca^2+^ transport, lipid synthesis, mitochondrial fission and fusion, oxidative and ER stress, autophagy, and inflammation [35]. ER stress has a significant impact on the mitochondria, including the regulation of mitochondrial homeostasis, fusion, and fission [25, 36, 37]. Under ER stress, the concentration of calcium ions transported from the ER to the mitochondria is increased, which can inhibit the expression of Mitofusin1 and 2 and block mitochondrial fusion. Noted, OPA1 and DRP1 can alter mitochondrial morphology through phosphorylation and dephosphorylation, and reduce mitochondrial fusion and mitochondrial number [38–41]. In turn, mitochondria function is also directly linked to ER stress. Elevated ATP production as a result of increased Ca^2+^ concentration in the mitochondria can help cells adapt to stressful environments. In effort to understand the molecular mechanism of LAMC2 on mitigating ER stress and maintaining normal mitochondrial function in tumor cells, IP-MS was used to detect LAMC2-binding proteins in A549 cells. As shown in our results, LAMC2 showed strong binding affinity to MYH9 and MYH10, which are subunits of non-myosin II. Fluorescence immunohistochemistry showed that LAMC2 co-localized with MYH9 and MYH10, and Co-IP experiments further demonstrated that LAMC2, MYH9 and MYH10 can physically interact with each other. Recent studies have found that MYH9 and MYH10 are closely related to the distribution and shape of mitochondria in cells. MYH9 has been previously shown to co-localize with the mitochondria [18]. Specifically, MYH9 was shown to bind STMP1, and that silencing MYH9 eliminated STMP1-induced DRP1 activation, mitochondrial division, and cell migration. The assembly of MYH10 and cytoskeleton can affect the cleavage process of mitochondria by interacting directly with the mitochondrial membrane, altering the distribution and shape of mitochondria within the cell [26, 42, 43]. The above results suggested that during ER stress, LAMC2 recruits mitochondria close to the ER via interacting with MYH9 and MYH10. In order to prove that ER stress promotes the formation of LAMC2/MYH9/MYH10 complexes, tunicamycin was used to treat tumor cells, and the results showed that the expression of LAMC2, MYH9, MYH10, GRP78 and DRP1 was significantly increased. Moreover, we showed that Tun-induced ER stress increased co-localization of LAMC2 with MYH9 and MYH10, as well as increased aggregation of interconnected ER and mitochondria at the perinuclear region. Silencing LAMC2 resulted in significantly lower levels of MYH9, MYH10, DRP1, and phosphorylated DRP1 proteins, and decreased aggregation of ER and mitochondria. Noted, DRP1 plays an important role in mitochondrial biosynthesis and quality control. DRP1 can increase ER-mitochondrial interactions by promoting the formation of tubular structures in the ER [21]. Under ER stress, protein kinase A (PKA) mediates phosphorylation of DRP1 to promote mitochondrial elongation, which enhances ER-mitochondrial associations. In order to clarify the relationship between DRP1 and LAMC2/MYH9/MYH10 complexes, the DRP1 inhibitor Mdivi-1 and Mdivi-1+Tun were used to treat tumor cells. Our results demonstrated that while Mdivi-1 had a slight effect on apoptosis, the combined treatment of Mdivi-1+Tun significantly induced apoptosis. More important, LAMC2 overexpression effectively inhibited Mdivi-1 and Mdivi-1+Tun- induced apoptosis.

As members of the LAMC2 ER stress response complex, MYH9 and MYH10 were also confirmed to be involved in the regulation of ER stress. In A549, MCF7, and MHCC-97H cells, silencing either MYH9 or MYH10 significantly increased ROS production, reduced mitochondrial membrane potential, and inhibited clonal formation and tumor cell viability. Moreover, silencing either MYH9 or MYH10 led to significant increases in ER stress-related protein levels, including that of GRP78, ATF6, PERK, and P-PERK. In addition, ER stress-induced decrease in BCL2 and increase in BAX, were compensated by LAMC2 overexpression. Furthermore, our data showed that MYH9 and MYH10 silencing significantly inhibited the co-localization between ER and mitochondria around the nucleus, leading to a reduction in DRP1 and phosphorylated DRP1 protein levels. MYH9 and MYH10 silencing were also shown to reverse the proliferative effects of LAMC2 overexpression on tumor cells. Collectively, these results provided strong indication that MYH9 and MYH10 are involved in LAMC2-induced ER stress response. Several studies have shown that MYH9 and MYH10 are involved in the development of tumors as oncogenes, which is consistent with the results of this study.

In conclusion, results from this study highlighted a novel mechanism for cancer cells to alleviate ER stress in order to proliferate in unfavorable cellular conditions. We showed that LAMC2 is localized to the ER of tumor cells. Moreover, we demonstrated that in order to combat ER stress and apoptosis, LAMC2 is elevated and forms protein complexes with MYH9 and MYH10 to promote ER-mitochondria interaction and mitochondria aggregation around the nucleus. Findings from this study suggested that LAMC2 can serve as a novel target for tumor therapy.

## Materials and methods

### Animal experiments

Three-week-old male BALB/c-nude mice were purchased from Yaokang Biotech. (Chengdu, China), and housed at Xi’an Jiaotong University Health Science Center- Central Laboratory of Animal, under standard conditions (pathogen-free, 23 ±2 °C, 40–70% humidity, ad libitum access to food and water, 12/12 hour (light/dark) cycle). The animals adjusted and were observed for one week before starting the experiments. 20 mice were randomly divided into two groups: Control and LAMC2. 1×10^6^ A549 cells suspended in 0.1 mL PBS were subcutaneously injected into the right dorsal part of each mouse. After one week later, when the tumors have emerged, each group was again randomly divided into two groups. Tunicamycin (0.02 mg/kg) (MedChemExpress, Shanghai, China) suspended in saline water was intravenously injected every 5 days. At day 34, the mice were sacrificed via CO_2_, and the tumors were extracted via standard surgery. Tumor size was measured using Vernier calipers. Tumor volume was calculated based on the volume=length x (width2)/2 formula. GraphPad Prism 8.0 (GraphPad Software) was used to generate all xenograft-related graphs. All animals were handled according to institutional guidelines, and all animal-related experiments were approved by the Institutional Animal Care and Use Committee of Xi’an Jiaotong University.

### Cell culture

Human lung adenocarcinoma A549 cells and breast cancer cell MCF7 were obtained from Genechem (Shanghai, Genechem Co. Shanghai, China). Human hepatoma cell line MHCC-97H were obtained from Botian (Xi’an, China). A549 cells were cultured in F12K (Hyclone) with 10% fetal bovine serum (FBS, Biological Industries, Israel). MCF7 and MHCC-97H cells were cultured in DMEM medium (Hyclone) with 10% fetal bovine serum (Biological Industries, Israel). Brefeldin A treatment: Cells were treated with BFA (MedChemExpress, Shanghai, China) dissolved in DMSO for 12 hr at various doses. Tunicamycin treatment: Cells were treated with Tunicamycin (MedChemExpress, Shanghai, China) dissolved in DMSO for 12 hr at various doses. Midiv-1 treatment: Cells were treated with Midiv-1 (MedChemExpress, Shanghai, China) dissolved in DMSO for 12 hr at various doses.

### Plasmid construction, siRNA synthesis, and cell transfection

siRNAs were designed and synthesized by GenePharma (Shanghai, China). Transfection was carried out using Polyplus jetPRIME (Polyplus, France) according to manufacturer’s protocol. Sequences of shRNA and siRNA are listed in Supplementary Table 1. LAMC2 his tagged (HG17053-CH), LAMC2 GFP tagged (HG17053-ACGLN) plasmids were purchased from Sino Biologica (Beijing, china). Construction of stable cell line: 293FT cells were transfected with LAMC2 GFP tagged plasmids and packaging vector; Lentivirus supernatant was collected 72 h after transfection; The lentiviral supernatant was filtered and infected cancer cells for 24 h. After infection, flow cytometry was used to detect the infection efficiency. Information on siRNAs used can be found in Supplementary Table 1.

### Western blotting

Total protein was extracted using RIPA lysis buffer containing phosphatase and proteinase inhibitor (Beyotime, Beijing, China), and protein concentrations were quantified using bicinchoninic acid (BCA) protein assay kit (Beyotime, Beijing, China). Whole-cell lysates were separated by 12% SDS-PAGE and transferred onto nitrocellulose membranes. The membranes were then blocked in 5% skim milk in TBST for 1 hour at room temperature, and incubated with primary antibodies overnight at 4 °C, followed by HRP-labeled secondary antibody for 1 h at room temperature. Protein bands were detected and analyzed using chemiluminescence and densitometric system. The complete list of primary and secondary antibodies used are listed in Supplementary Table 2.

### Cell activity

Cells (5 × 10^3^) were plated into 96-well plates and cultured for 48 h. Cell activity was analyzed using CellTiter-Lumi™ luminescent cell viability assay kit (Beyotime, Beijing, China). A microplate reader (POLARstar OPTIMA, BMG, Germany) was used to analyze absorbance at 570 nm.

### Live-cell fluorescence detection

The cells were seeded into cell culture plates and stained with JC1 staining solution (40705ES03, Yeasen Biotechnology, Shanghai, China) following the instruction manual. The fluorescence was observed with Leica DMi8 fluorescence microscope. Reactive Oxygen Species were detected using the Reactive Oxygen Species Assay Kit (50101ES01, Yeasen Biotechnology, Shanghai, China), and visualized with Leica DMi8 fluorescence microscope. The quantification of fluorescence results was done using the Image J software. Additionally, ER-Tracker Green (BODIPY FL Glibenclamide) (40763ES20, Yeasen Biotechnology, Shanghai, China), ER-Tracker Blue-White DPX (40761ES50, Yeasen Biotechnology, Shanghai, China) and MitoTracker Red CMXRos (40741ES50, Yeasen Biotechnology, Shanghai, China) were used to stain the endoplasmic reticulum or mitochondria of live cells. The staining was visualized using laser scanning confocal microscopy (Leica, TCS SP8 DIVE, Germany), and the co-localization was quantified using the co-localization Finder plugin of ImageJ software. The experimental protocols were conducted as per the standards.

### Flow cytometry

Cells were trypsinized and resuspended in 1X binding buffer, and stained using the Annexin-V-FITC/PI Apoptosis Detection kit (40305ES50, Yeasen Biotechnology, Shanghai, China). The cells were then washed with PBS, and cell apoptosis was analyzed using flow cytometry (FACSCalibur, BD, Biosciences, USA). Mitochondrial membrane potential detection: Cells were tripsinized and resuspended in 1X binding buffer, and stained using the JC1 staining solution (40705ES03, Yeasen Biotechnology, Shanghai, China) following the instruction manual. The cells were re-suspended with fresh culture solution for subsequent flow cytometry analysis. Reactive oxygen species (ROS) detection: DCFH-DA (50101ES01,Yeasen Biotechnology, Shanghai, China)was diluted in serum-free medium at 1:1000 to a final concentration of 10 μM; Cells were tripsinized and resuspended in appropriate volume of diluted DCFH-DA working solution. After staining, the cells were re-suspended with fresh serum-free medium and immediately detected by flow cytometry.

### Detection of mitochondrial calcium

Cells were transfected with si-LAMC2, si-MYH9, si-MYH10, or control. After 24 hours, each group of cells were treated with Tun at different time points (0, 0.5, 1, 1.5, 2, 3, 4, 5, and 7 h). Rhod-2-AM, Cell Permeable kit (40776ES72, Yeasen Biotechnology, Shanghai, China) was then utilized to detect the mitochondrial calcium at 578 nm absorbance using a microplate reader (POLARstar OPTIMA, BMG, Germany). To obtain the relative fluorescence fold change, the fluorescence values of each Tun treatment group were normalized by the 0-hour treatment group. Finally, GraphPad Prism 8.0 was utilized to calculate the relative fluorescence change curve and the area under the curve.

### Colony formation

Cells were trypsinized and seeded in 6-well plates (500 cells/well), and cultured for 10–14 days. Afterwards, the cells were stained with 0.1% crystal violet solution (Sigma-Aldrich, USA) for 15 min, and colony numbers were evaluated using Quantity One Software (Bio-Rad, USA).

### Immunohistochemical staining

Formalin-fixed tissue samples were cut into 3um thickness. Samples were deparaffinized using 100% xylene and hydrated using graded ethanol (100%, 95%, 80%, 70%, 50%, and 30%). 0.01 M citrate buffer (pH 6.0) was used for antigen removal, and the samples were then incubated with primary antibodies (LAMC2, GRP78, and Ki67) at 4 °C overnight. Immunodetection was performed using 3,3’-diaminobenzidine (DAB, Dako) and hematoxylin staining according to manufacturer’s instructions the following day. Images were obtained with Leica Microsystems (Leica, Germany). Intensity was manually scored. High expression was determined when more than 50% positive staining cells were detected in five randomly selected fields.

### Immunofluorescence microscopy

Cells were fixed with 4% paraformaldehyde for 20 min, washed 3 times with PBS, followed by blocking using 10% normal goat serum in PBS (0.2% Triton X-100) at room temperature for 1 hr. Cells were then incubated with primary antibodies (LAMC2, MYH9, MYH10, DRP1) overnight at 4 °C, and fluorochrome-conjugated secondary antibody for 1 h at room temperature in the dark. After washing with PBS, 4′6-diamidino-2-phenylindole (DAPI) (0.1 mg/mL) (Beyotime, Beijing, China) was added. Immunofluorescent signals were detected using a fluorescence microscope (Leica, TCS SP8 DIVE, Germany).

### Co-IP assays

Total protein from cells were extracted using lysis buffer (150 mM NaCl, 1% NP-40, 50 mM Tris-HCl, pH8.0, protease inhibitor). Lysates were centrifuged at 13,000 x g at 4 °C for 10 min. Total cell lysates were then incubated with primary antibodies (anti-LAMC2, anti-MYH9, anti-MYH10, anti-His) on Dynal magnetic beads (Invitrogen, CA, USA) overnight at 4 °C. The beads were then washed five times with PBS, and the immunoprecipitates were used for western blotting analysis.

### Bioinformatics analysis

FPKM transcriptome expression matrix data were downloaded from UCSC XENA for 33 tumors; The Human Gene Annotation GTF file (V43) was Download from GENCODE; Human and mouse mitochondrial-related gene files and pathway reference files for GSEA clustering were download from MitoCartaP.L. For Hallmark GSEA analysis using the clusterProfiler package, refer to H.ARI.v7.5.1.entrez.GMT; Mitochondrial GSEA pathway analysis was conducted using the clusterProfiler package, refer to MitoPathway 3.0.gmx. Cancer cell gene expression data was downloaded from CCLE (Cancer Cell Line Encyclopedia), and the Drug sensitivity AUC (CTD^2) data of 7 chemotherapeutic drugs were downloaded from PRISM (profiling relative inhibition simultaneously in mixtures). The correlation between drug sensitivity evaluation index AUC and gene expression was analyzed by Prism 8.0 (GraphPad Software).

### Statistics

All experiments were repeated three independent times. Graphical data presented as mean ± standard deviation. Student’s t-test was used to determine the difference between two independent groups. p values < 0.05 were considered statistically significant. GraphPad Prism 8.0 (GraphPad Software) was used for all data analyses.

### Supplementary information

Supplementary Figures

Supplementary Tables

IP-MS identified peptide list

IP-MS identified protein table

ARRIVE guidelines

## Data Availability

All data generated or analyzed during this study are available from the corresponding author on reasonable request
