# Supplementary material for: LAMC2 mitigates ER stress by enhancing ER-mitochondria interaction via binding to MYH9 and MYH10
Source: Cancer Gene Ther. 2023 Oct 27;31(1):43–57. doi: 10.1038/s41417-023-00680-5 (PMC10794146; doi:10.1038/s41417-023-00680-5)
Supplement: Supplementary file 1 — Supplementary Figures [file 41417_2023_680_MOESM1_ESM.docx]

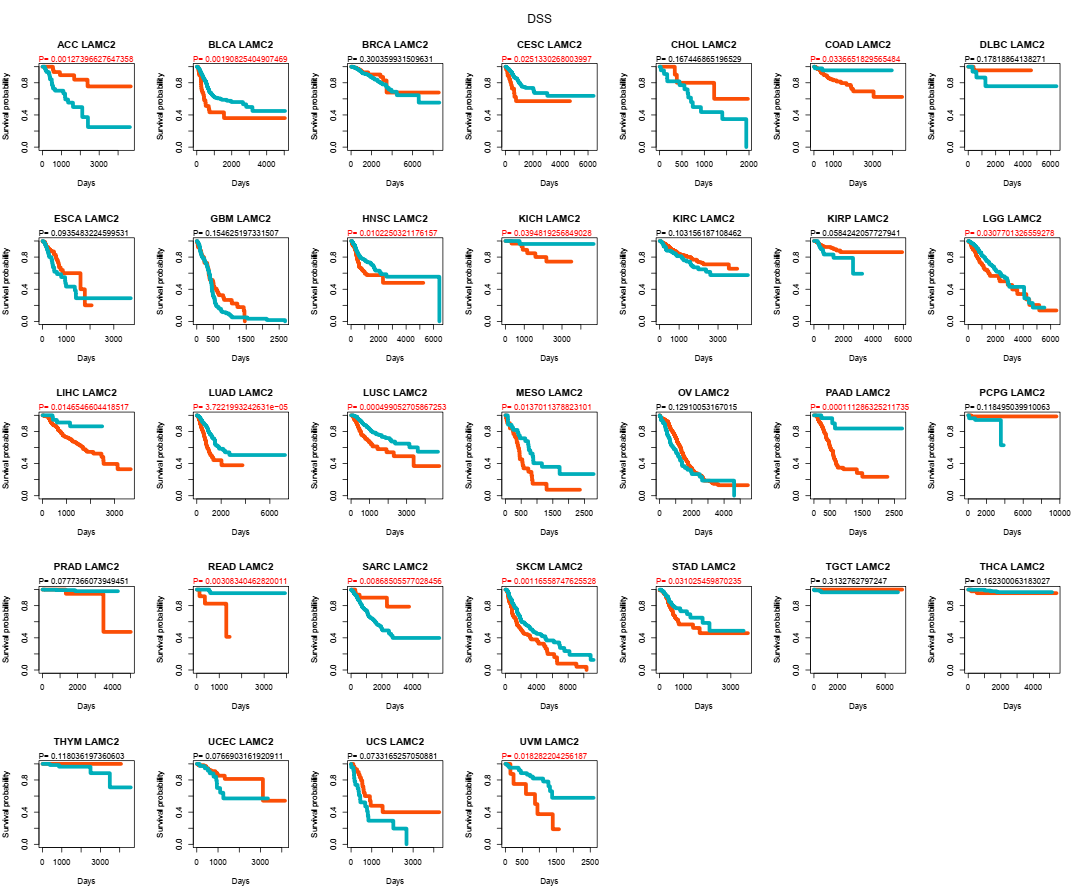

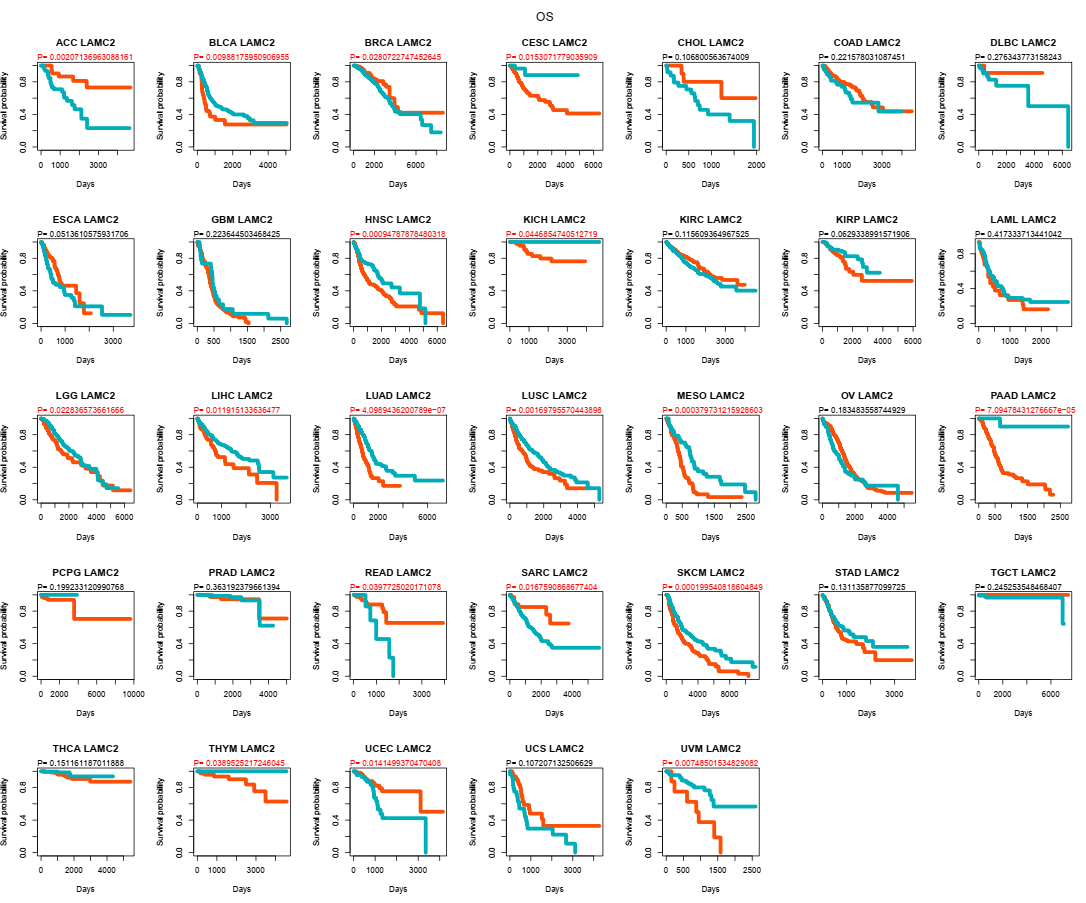

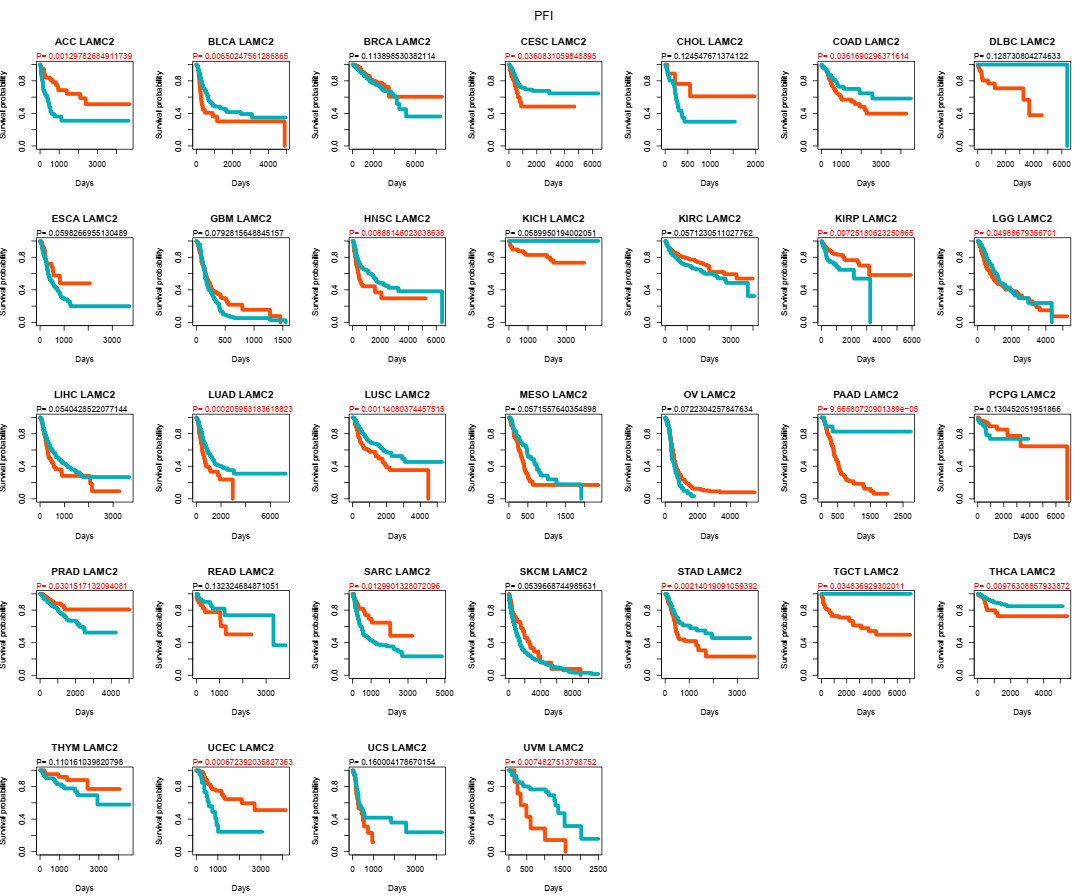

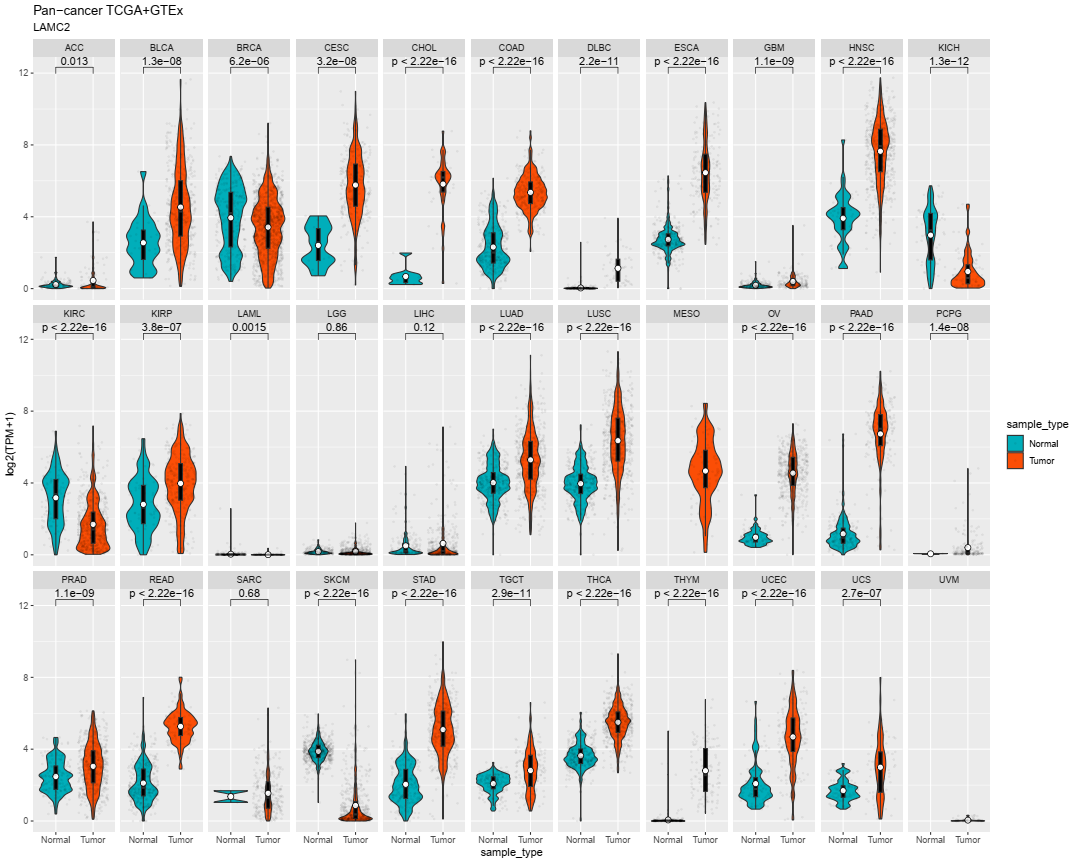


D

C

A

B

***Supplementary Figure 1***

1. TCGA data analysis of LAMC2 expression in 33 types of tumor tissues and corresponding controls.
2. TCGA data analysis of the correlation between LAMC2 expression and overall survival in 33 different cancer types.
3. TCGA data analysis of the correlation between LAMC2 expression and progression free interval in 33 different cancer types.
4. TCGA data analysis of the correlation between LAMC2 expression and disease specific survival in 33 different cancer types.


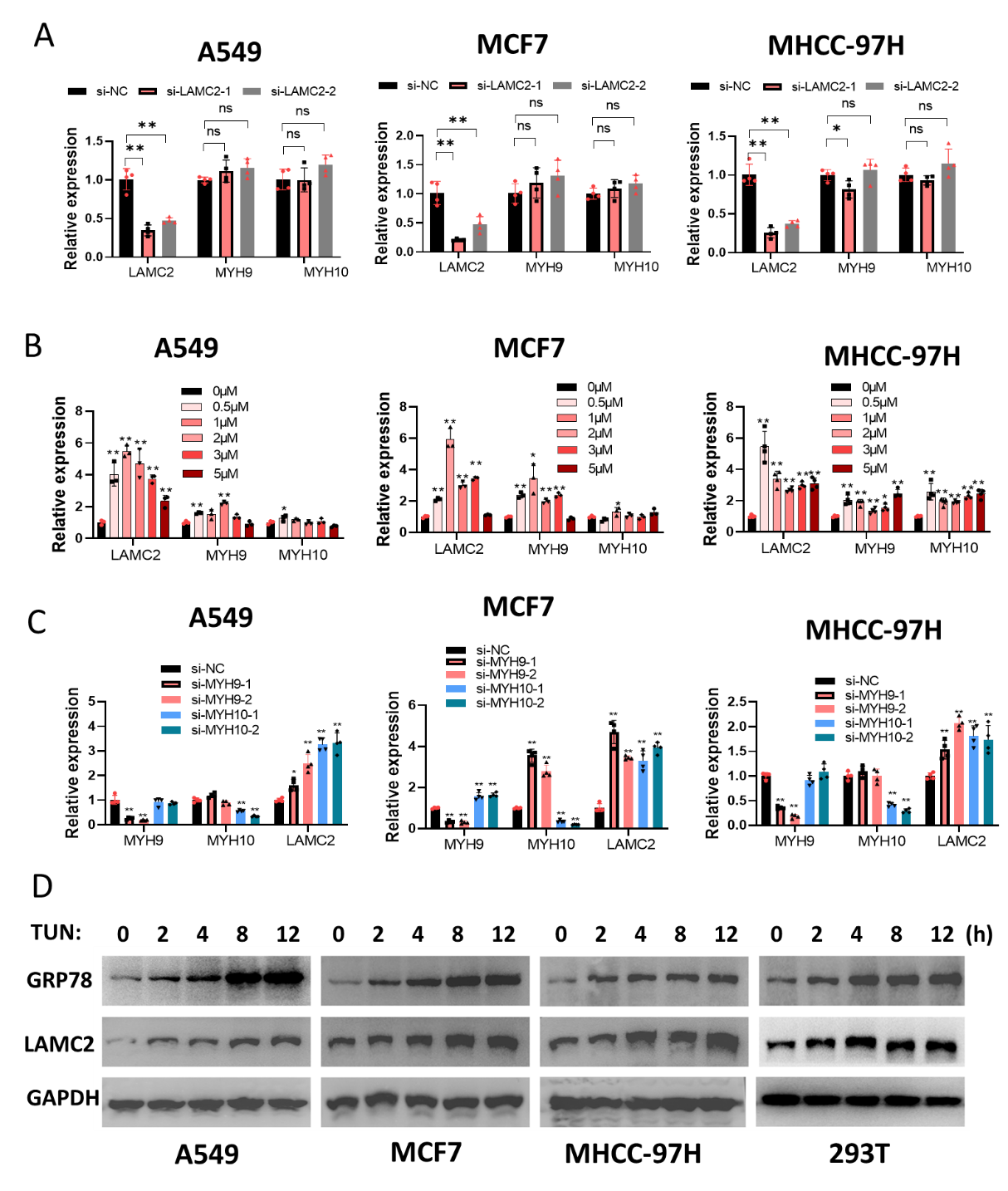


***Supplementary Figure 2***

1. qRT-PCR analysis of LAMC2, MYH9, and MYH10 mRNA levels in si-LAMC2 cancer cells.
2. qRT-PCR analysis of LAMC2, MYH9, and MYH10 mRNA levels in cancer cells treated with various doses of Tun.
3. qRT-PCR analysis of LAMC2, MYH9, and MYH10 mRNA levels in si-MYH9 and si-MYH10 cancer cells. **p < 0.05, **p < 0.01.*
4. Western blot analysis of LAMC2 and GRP78 in response to TUN treatment (0.5μM) at various time points.


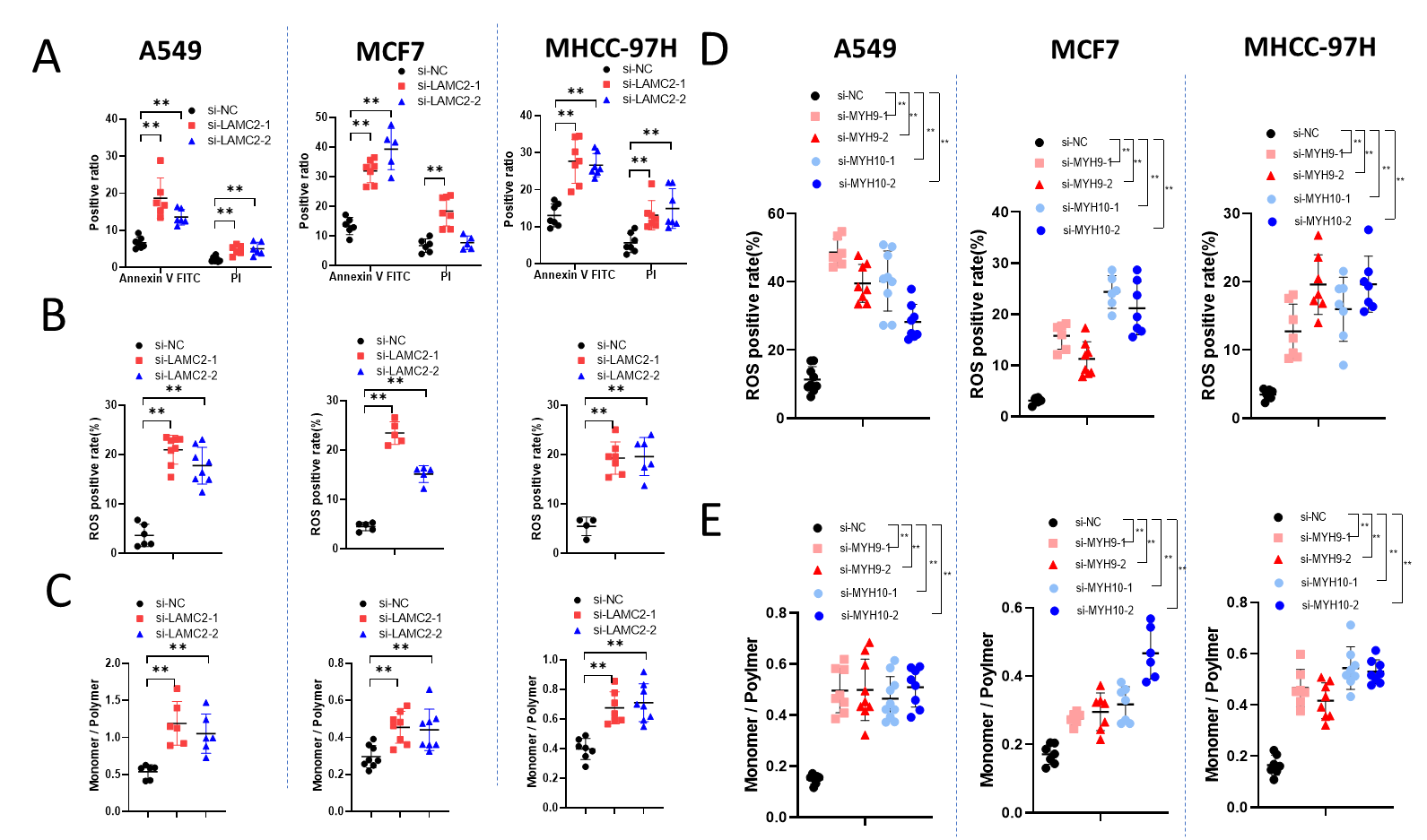


***Supplementary Figure 3***

1. Quantification of relative Annexin V/FITC and PI ratio in LAMC2 knockdown cancer cells.
2. Quantification of ROS positive rate (%) in LAMC2 knockdown cancer cells.
3. Quantification of monomer/polymer ratio in LAMC2 knockdown cancer cells.
4. Quantification of ROS positive rate (%) in MYH9 and MYH10 knockdown cancer cells.
5. Quantification of monomer/polymer ratio in MYH9 and MYH10 knockdown cancer cells.


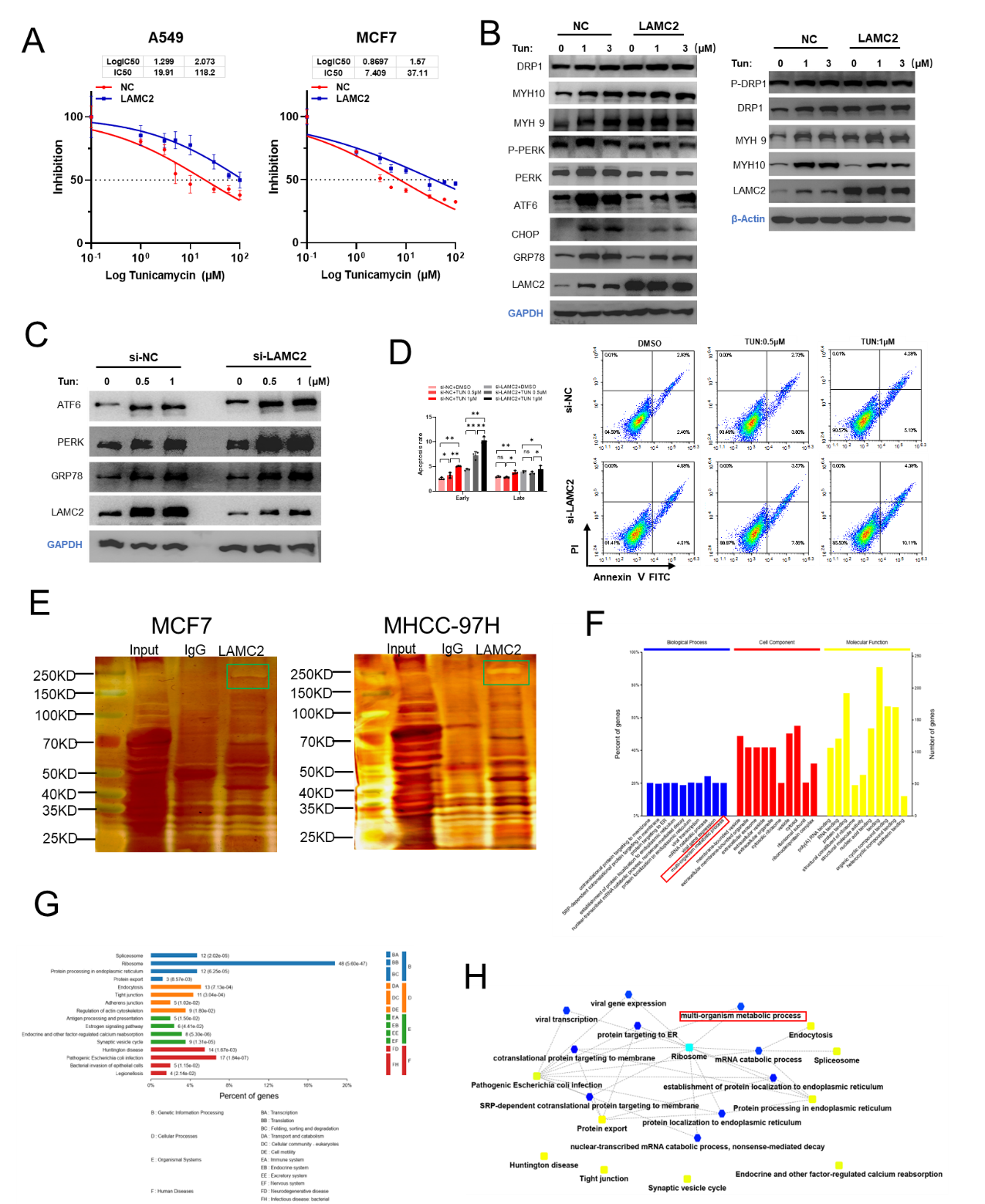


***Supplementary Figure 4***

1. The 50% inhibitory concentration of LAMC2 overexpressing A549 and MCF7 cells treated with Tun.
2. Western blots of DRP1, MYH10, MYH9, P-PERK, PERK, ATF6, CHOP, GRP78, LAMC2, GAPDH, and B-actin in control and LAMC2 overexpressing MHCC-97H cells treated with Tun.
3. Western blots of LAMC2, GRP78, PERK, ATF6 and GAPDH in control and LAMC2 knockdown A549 cells treated with Tun.
4. Flow cytometry analysis of apoptosis in control and LAMC2 knockdown A549 cells treated with Tun.
5. Silver staining image of proteins co-purified with LAMC2 in MCF7 and MHCC-97H cells.

(F-H) GO (F), KEGG (G), and protein-protein interaction (H) analyses of top biological processes correlated with LAMC2 interacting proteins.

*Supplementary Figure 5*

Bioinformatics analysis of the relationship between LAMC2 and the proliferation of tumor and non-tumor cells (Mann Whitney test, *P*＜0.05). The data were downloaded from the Depmap database(https://depmap.org/portal/), and according to the database, a lower Effect score indicates a higher level of dependence.
