# Supplementary material for: LAMC2 mitigates ER stress by enhancing ER-mitochondria interaction via binding to MYH9 and MYH10
Source: Cancer Gene Ther. 2023 Oct 27;31(1):43–57. doi: 10.1038/s41417-023-00680-5 (PMC10794146; doi:10.1038/s41417-023-00680-5)
Supplement: Supplementary file 2 — Supplementary Tables [file 41417_2023_680_MOESM2_ESM.docx]

**Supplementary Tables**

Table 1. si-RNA information

| Name | Sequence |
| --- | --- |
| Negative siRNA (NC-siRNA) | 5'-TTCTCCGAACGTGTCACGT-3' |
| si-LAMC2-1 | 5'-GAAGCTTCCTTGGGAAACA-3 |
| si-LAMC2-2 | 5'-GTCAAAGCCTGTCCTTTGA-3' |
| si-MYH9-1 | 5'-CCGTACAACAAATACCGCT-3' |
| si-MYH9-2 | 5'-GGGTATCAATGTGACCGAT-3' |
| si-MYH10-1 | 5'-GAGATTCTGTCAATGCTTA-3' |
| si-MYH10-2 | 5'-CAAGTAAGCTGCAGAATGA-3' |

Table 2. Antibody information

| Antibody | Company | Catalog No. |
| --- | --- | --- |
| LAMC2 | Abcam | ab210959 |
| MYH9 | Proteintech | 11128-1-AP |
| MYH10 | Abcam | ab230823 |
| GRP78 | Proteintech | 11587-1-AP |
| ATF6 | Proteintech | 66563-1-Ig |
| PERK | Proteintech | 24390-1-AP |
| P-PERK | Proteintech | 29546-1-AP |
| CHOP | Proteintech | 15204-1-AP |
| DRP1 | Proteintech | 12957-1-AP |
| P-DRP1 | Cell Signaling Technology | #3455 |
| BAX | Proteintech | 0599-2-Ig |
| His | Proteintech | 66005-1-Ig |
| GFP | abways | AB0005 |
| Ki67 | Proteintech | 27309-1-AP |
| BCL2 | Cell Signaling Technology | #15071 |
| PARP | Cell Signaling Technology | #9532 |
| Cleaved PARP | Cell Signaling Technology | #9548 |
| Csepase6 | Cell Signaling Technology | #9762 |
| Cleaved Caspase-6 | Cell Signaling Technology | #9761 |
| Csepase9 | Cell Signaling Technology | #9504 |
| Cleaved Caspase-9 | Cell Signaling Technology | #9509 |
| GAPDH | abways | AB0038 |
| β-Actin | abways | AY0573 |
| Goat Anti-Rabbit IgG | abways | AB0101 |
| Goat Anti-Mouse IgG | abways | AB0102 |

Table 3. Primer information

| Gene | Strand | Sequence |
| --- | --- | --- |
| LAMC2 | Forward | TACCAGAGCCAAGAACGCTG |
|  | reverse | CTACACTGAGAGGCTGGTCCAT |
| MYH9 | Forward | CATGGAGGCCGAGATGATCC |
|  | reverse | TAACGCCAGGGCTCCTTTG |
| MYH10 | Forward | GAGCCACCAGTGGACCGT |
|  | reverse | ACGGTACGAAACATGCCCTT |
| GAPDH | Forward | GTAGAGGCAGGGATGATGTTC |
|  | reverse | GCCAAAAGGGTCATCATCTC |
